# Serum pentraxin-3 in patients with chronic obstructive pulmonary disease: A meta-analysis

**DOI:** 10.17305/bb.2024.10875

**Published:** 2024-12-01

**Authors:** Yan Zhu, Chongyang Wang

**Affiliations:** 1 Department of Emergency Medicine, Shaoxing Seventh People’s Hospital, Shaoxing, China

**Keywords:** Chronic obstructive pulmonary disease (COPD), pentraxin-3 (PTX-3), biomarker, acute exacerbation, meta-analysis

## Abstract

The association between serum pentraxin-3 (PTX-3) levels and chronic obstructive pulmonary disease (COPD) has been explored in several studies. However, the results remain inconsistent. This meta-analysis aims to evaluate the differences in serum PTX-3 levels between COPD patients and healthy controls, as well as between patients with acute exacerbations of COPD (AECOPD) and stable COPD. Databases including PubMed, Embase, Web of Science, Wanfang, and China National Knowledge Infrastructure (CNKI) were systematically searched. A random-effects model was used to pool the results, accounting for the potential impact of heterogeneity. Subgroup and meta-regression analyses were performed to evaluate the influence of study characteristics on the outcome. The initial search identified 274 articles, with 17 studies meeting the inclusion criteria. These studies included a total of 996 AECOPD patients, 1414 stable COPD patients, and 1016 healthy controls. The meta-analysis showed significantly higher serum PTX-3 levels in COPD patients compared to healthy controls (standardized mean difference [SMD]: 0.51, 95% confidence interval [CI]: 0.30–0.73, *P* < 0.001; *I*^2^ ═ 85%). Subgroup and meta-regression analyses suggested that the results were not significantly affected by the age, sex, or smoking status of the patients. Additionally, serum PTX-3 levels were higher in AECOPD patients compared to stable COPD patients (SMD: 0.58, 95% CI: 0.41–0.74, *P* < 0.001; *I*^2^ ═ 59%). In conclusion, serum PTX-3 levels are elevated in COPD patients, particularly during acute exacerbations, compared to stable COPD patients and healthy controls. PTX-3 may serve as a potential biomarker for COPD severity and exacerbation status.

## Introduction

Chronic obstructive pulmonary disease (COPD) is one of the leading causes of morbidity and mortality worldwide, affecting over 250 million individuals and resulting in approximately three million deaths annually [1, 2]. Pathophysiologically, COPD is characterized by persistent respiratory symptoms and airflow limitation due to airway and/or alveolar abnormalities, usually caused by significant exposure to noxious particles or gases, most commonly from smoking [3, 4]. Despite advances in treatment, the prognosis of patients with COPD remains poor, especially for those experiencing frequent acute exacerbations of COPD (AECOPD), which accelerate disease progression, diminish quality of life, and increase the risk of mortality [5, 6]. Therefore, identifying reliable biomarkers for COPD and its exacerbations is crucial for improving patient management and outcomes [7].

Biomarkers play a critical role in the diagnosis, monitoring, and prognosis of various diseases, including COPD [8, 9]. In the context of COPD, biomarkers can provide insights into the disease’s pathogenesis, identify patients at risk of exacerbations, and guide therapeutic decisions [10]. Acute exacerbations, often triggered by infections or environmental pollutants, significantly contribute to the overall disease burden in COPD patients [11]. Pentraxin-3 (PTX-3), an acute-phase protein, has emerged as a promising candidate due to its role in the innate immune response and inflammation, both of which are pivotal in COPD pathogenesis and exacerbations [12, 13]. PTX-3, a protein belonging to the pentraxin family, is produced by various cells, including endothelial cells, macrophages, and dendritic cells, in response to inflammatory stimuli [14]. It plays a critical role in regulating immune responses and inflammation, which are fundamental processes in the pathophysiology of COPD [15, 16]. Recent studies have investigated the relationship between serum PTX-3 levels and COPD, but the findings have been inconsistent, with some studies reporting elevated levels in COPD patients [17–25] and others finding no significant differences [26–31]. These discrepancies may be due to variations in study design, population characteristics, and measurement techniques. In view of the uncertainty, this meta-analysis aims to synthesize existing evidence to clarify the role of PTX-3 in COPD and its exacerbations, providing a comprehensive assessment of its potential as a biomarker for disease severity and exacerbation status.

## Materials and methods

The Preferred Reporting Items for Systematic Reviews and Meta-Analyses (PRISMA 2020) [32, 33] and the Cochrane Handbook for Systematic Reviews and Meta-Analyses [34] were followed in this meta-analysis during study design, data collection, statistical analysis, and result interpretation.

### Inclusion and exclusion criteria

Studies fulfilled the following criteria were included:

(1) *Population:* Studies involving patients diagnosed with COPD and healthy control subjects; COPD patients with other cardiopulmonary diseases, such as coronary artery disease, heart failure, asthma, and lung cancer, were excluded.
(2) *Intervention and comparison:* Studies comparing serum PTX-3 levels between COPD patients and healthy controls, as well as studies comparing serum PTX-3 levels between patients with AECOPD and those with stable COPD.
(3) *Outcome:* Studies reporting quantitative measurements of serum PTX-3 levels, including the mean levels and standard deviation (SD); or where these data could be estimated.
(4) *Design:* Observational studies, including cross-sectional, cohort, and case-control studies published in peer-reviewed journals.
(5) *Language:* Articles published in English or Chinese.
(6) *Time Frame:* No restrictions on the publication date.

The exclusion criteria included:

(1) Studies that do not include a control group of healthy subjects or do not differentiate between AECOPD and stable COPD.
(2) Non-original research articles such as reviews, meta-analyses, case reports, editorials, letters, conference abstracts, and animal studies.
(3) Studies with insufficient data for extraction or ambiguous reporting of serum PTX-3 levels.
(4) Studies not published in English or Chinese, or not published as full-length articles in peer-reviewed journals.

### Literature search

A comprehensive and systematic literature search was conducted across PubMed, Embase, Web of Science, Wanfang, and China National Knowledge Infrastructure (CNKI) databases from their inception to May 10, 2024. The search strategy involved the combination of the following terms: (“pentraxin-3” OR “pentraxin 3” OR “PTX-3”) AND (“chronic obstructive pulmonary disease” OR “COPD” OR “chronic obstructive lung disease” OR “chronic obstructive airway disease” OR “emphysema” OR “chronic airflow limitation” OR “chronic airway obstruction”). The search was limited to studies in humans. In addition, the reference lists of identified articles were manually searched to locate further relevant studies.

### Study quality evaluation and data extraction

The quality of included studies was assessed using the Newcastle–Ottawa Scale (NOS), which evaluates studies based on three domains: selection of cases and controls, comparability of groups, and ascertainment of the exposure or outcome. Each study could receive a maximum of nine points, with studies scoring 6 or more considered high quality. Two independent reviewers performed the quality assessment, and any discrepancies were resolved through discussion between the two authors to reach a consensus. Additionally, two independent reviewers extracted data from the selected studies. The extracted data included: (1) study characteristics (first author, publication year, country, study design); (2) participant information: sample size of patients with AECOPD, stable COPD, and healthy controls, age and sex of the subjects, and smoking status of patients with COPD; (3) techniques used to measure serum PTX-3 levels, such as enzyme-linked immunosorbent assay (ELISA); and (4) outcome data: mean and SD of serum PTX-3 levels in healthy controls, AECOPD, and stable COPD groups.

### Ethical statement

Ethical approval was not required for this study in accordance with local/national guidelines. Written informed consent to participate in the study was not required in accordance with local/national guidelines.

### Statistical analysis

The primary objective was to evaluate the difference in serum PTX-3 levels between patients with COPD and healthy controls, while the secondary objective was to evaluate the difference in serum PTX-3 levels between patients with AECOPD and stable COPD. For studies that included patients with AECOPD, stable COPD, and healthy controls, two separate comparisons were made: one between patients with AECOPD and controls, and another between patients with stable COPD and controls. The shared control groups were equally split and included as independent comparisons to overcome a unit-of-analysis error, according to the instructions of the Cochrane Handbook [34]. The difference in serum PTX-3 levels was summarized as standardized mean difference (SMD) and corresponding 95% confidence interval (CI) because different ELISA kits were used among the included studies and the level of serum PTX-3 was reported in different units. Cochrane *Q* test and *I*^2^ statistics were used to estimate study heterogeneity [35], with significant statistical heterogeneity reflected by an *I*^2^ > 50%. The results were combined using a random-effects model incorporating heterogeneity’s influence [34]. For outcomes involving at least ten comparisons, predefined subgroup analyses were also performed to evaluate the influences of study characteristics on the outcome. The medians of the continuous variables were used as the cutoffs for defining subgroups. Additionally, a meta-regression analysis was performed to evaluate the influence of study characteristics in continuous variables on the outcome of the meta-analysis, such as mean age, proportion of men, proportion of smokers in patients with COPD, and the study quality scores. To estimate publication bias in the meta-analysis, funnel plots were constructed and visually inspected for symmetry [36]. Additionally, Egger’s regression test was performed to further assess the presence of publication bias [36]. The statistical analysis was carried out using RevMan (Version 5.1; Cochrane Collaboration, Oxford, UK) and Stata software (version 12.0; Stata Corporation, College Station, TX, USA). A two-sided *P* < 0.05 suggests statistical significance.

## Results

### Results of literature search

**Figure 1.**
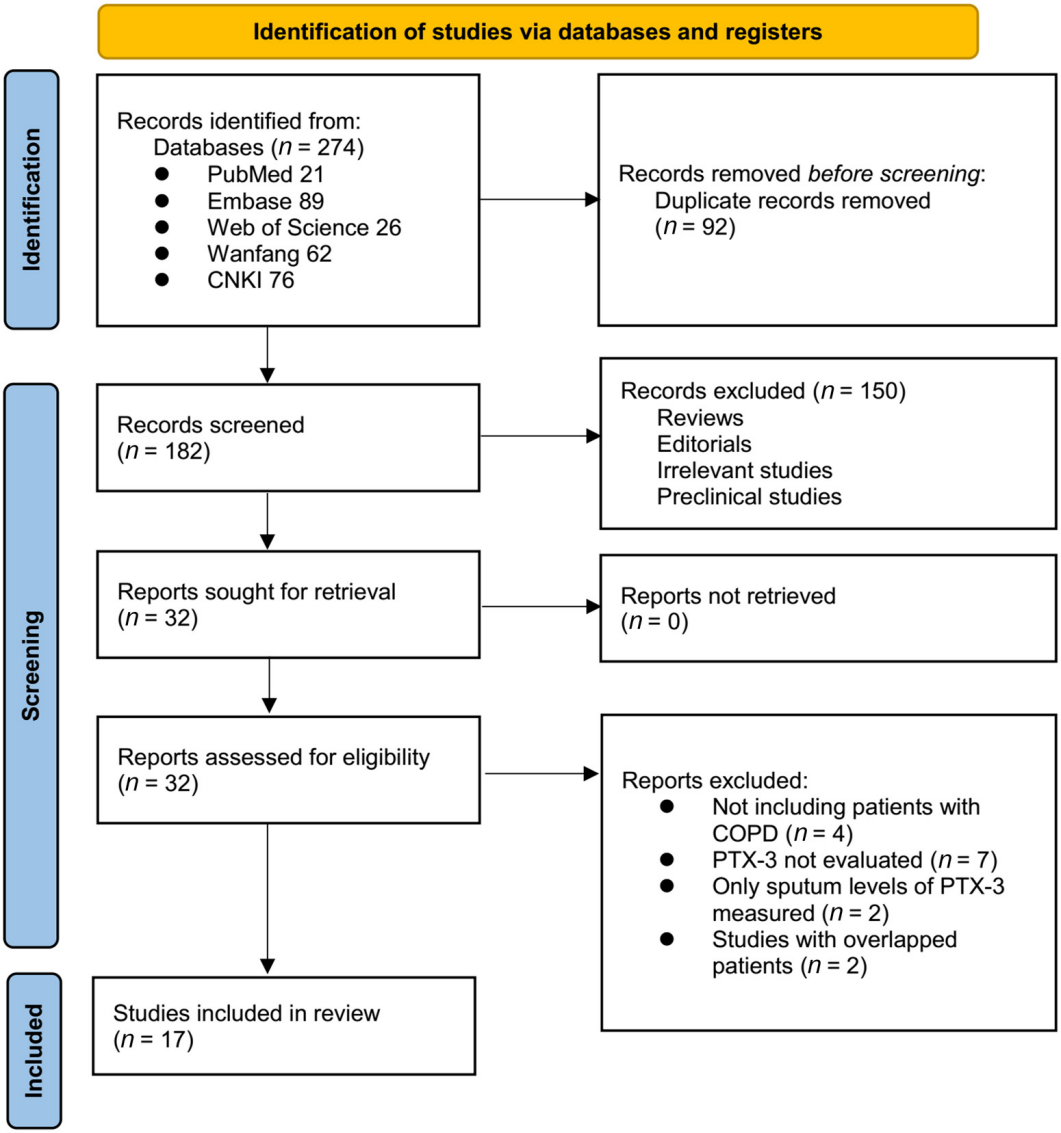
**The flowchart depicts the process of database search and study inclusion.** CNKI: China National Knowledge Infrastructure; COPD: Chronic obstructive pulmonary disease; PTX-3: Pentraxin-3.

The process of study inclusion is presented in Figure 1. The initial database search identified 274 articles. After removing duplicates, 182 articles remained. Screening of titles and abstracts further excluded 150 articles, mostly because these studies were not relevant to the aim of the meta-analysis, leaving 32 for full-text review. Of these, 15 articles were excluded for reasons specified in Figure 1. Finally, 17 case-control studies [17–31, 37, 38] met the inclusion criteria for the meta-analysis.

### Overview of study characteristics

Table 1 presents the summarized characteristics of the included studies. Overall, 17 case-control studies [17–31, 37, 38], published between 2012 and 2023 and performed in Belgium, Italy, Turkey, China, and the United Kingdom, were included in the meta-analysis. A total of 996 patients with AECOPD, 1414 patients with stable COPD, and 1016 healthy controls were involved in these studies. The mean ages of the subjects ranged from 57 to 69 years, and the proportions of men ranged from 43% to 85%. The serum levels of PTX-3 were all measured with ELISA using different commercially available kits. Potential confounding factors, such as age, sex, body mass index (BMI), and smoking status, were matched to different degrees between groups. The NOS of the included studies ranged from six to nine stars, suggesting overall good study quality (Table 2).

**Table 1:** Characteristics of the included studies

**Study**	**Location**	**Study design**	**No. of patients with AECOPD**	**No. of patients with stable COPD**	**No. of healthy control**	**Mean age (years)**	**Men (%)**	**Smokers in COPD**	**Methods for measuring serum PTX-3**	**Variables matched or adjusted**
Van Pottelberge, 2012	Belgium	CC	0	33	27	62.2	85	100	ELISA	Age and sex
Beghe, 2013	Italy	CC	0	70	24	68.2	71.3	100	ELISA	Age, sex, BMI, and smoking
Duran, 2015	Turkey	CC	126	0	48	66.1	79.9	85.7	ELISA	Age and BMI
Kurt, 2015	Turkey	CC	0	54	31	57.6	77.6	83.3	ELISA	None
Li, 2016	China	CC	0	110	110	67	78.2	58	ELISA	Age, sex, and BMI
Thulborn, 2017	UK	CC	142	142	0	69	72	96	ELISA	Age, sex, BMI, and smoking
Huang, 2017	China	CC	90	90	90	66.3	67	NR	ELISA	Age and sex
He, 2018	China	CC	23	114	0	62.6	83.9	84.7	ELISA	Age, sex, and smoking
Chou, 2018	China	CC	55	0	25	63	43.8	NR	ELISA	Age and sex
Ye, 2018	China	CC	70	70	70	68.6	62.9	100	ELISA	Age, sex, and smoking
Qiu, 2018	China	CC	135	0	75	57.2	75.8	66.5	ELISA	Age
Poznanski, 2019	China	CC	0	17	17	67	47.1	54.5	ELISA	None
Wu, 2020	China	CC	57	62	60	58.5	55.9	25.6	ELISA	Age, sex, BMI, and smoking
Huang, 2020	China	CC	45	60	35	59.8	67.9	80	ELISA	Age and sex
Xu, 2022	China	CC	0	127	73	59.3	56.5	75.6	ELISA	Age, sex, BMI, and smoking
Gu, 2023	China	CC	173	285	71	66.6	79.4	78.6	ELISA	Sex
Liu, 2023	China	CC	80	180	260	64.3	66.5	73.8	ELISA	Age, sex, BMI, and smoking

COPD: Chronic obstructive pulmonary disease; AECOPD: Acute exacerbated chronic obstructive pulmonary disease; PTX-3: Pentraxin-3; CC: Case-control studies; NR: Not reported; ELISA: Enzyme-linked immunosorbent assay; BMI: Body mass index.

**Table 2:** Study quality evaluation via the Newcastle–Ottawa Scale

	**Adequate definition of the cases**	**Representativeness of the cases**	**Selection of controls**	**Definition of controls**	**Controlled for age**	**Controlled for other confoundings**	**Ascertainment of the exposure**	**Same method of ascertainment of exposure for cases and controls**	**Non-response rate**	**Overall**
Van Pottelberge, 2012	1	0	1	1	1	0	1	1	1	7
Beghe, 2013	1	0	1	1	1	1	1	1	1	8
Duran, 2015	1	0	1	1	1	1	1	1	1	8
Kurt, 2015	1	0	1	1	0	0	1	1	1	6
Li, 2016	1	0	1	1	1	1	1	1	1	8
Thulborn, 2017	1	0	1	1	1	1	1	1	1	8
Huang, 2017	1	0	1	1	1	1	1	1	1	8
He, 2018	1	1	1	1	1	1	1	1	1	9
Chou, 2018	1	0	1	1	1	1	1	1	1	8
Ye, 2018	1	0	1	1	1	1	1	1	1	8
Qiu, 2018	1	1	1	1	1	0	1	1	1	8
Poznanski, 2019	1	0	1	1	0	0	1	1	1	6
Wu, 2020	1	0	1	1	1	1	1	1	1	8
Huang, 2020	1	0	1	1	1	0	1	1	1	7
Xu, 2022	1	0	1	1	1	1	1	1	1	8
Gu, 2023	1	1	1	1	0	0	1	1	1	7
Liu, 2023	1	0	1	1	1	1	1	1	1	8

### Difference in serum PTX-3 levels between patients with COPD and controls

**Figure 2.**
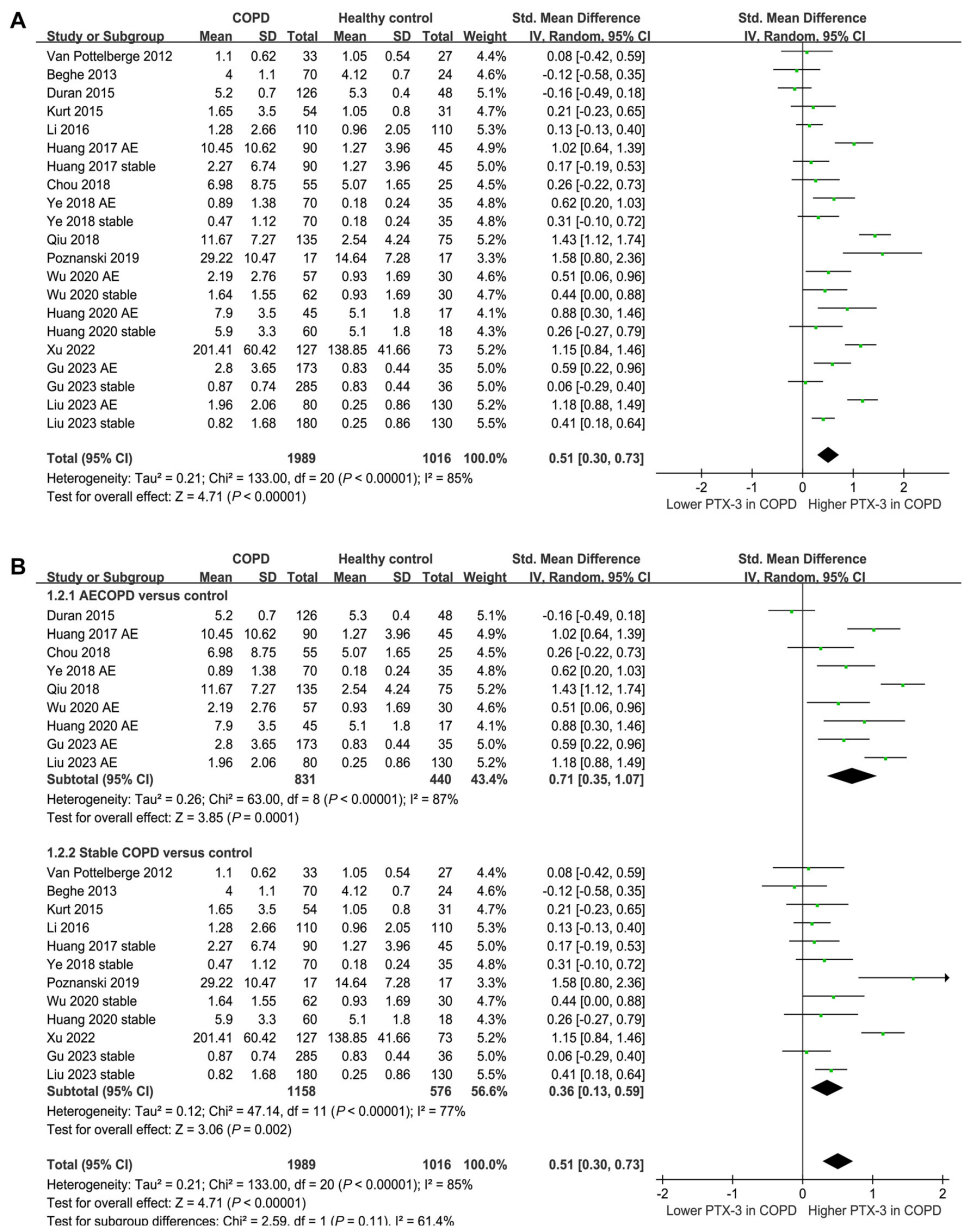
**Forest plots for the meta-analysis comparing serum PTX-3 between patients with COPD and healthy controls; (A) overall meta-analysis; (B) subgroup analysis according to disease status of COPD.** PTX-3: Pentraxin-3; COPD: Chronic obstructive pulmonary disease.

**Figure 3.**
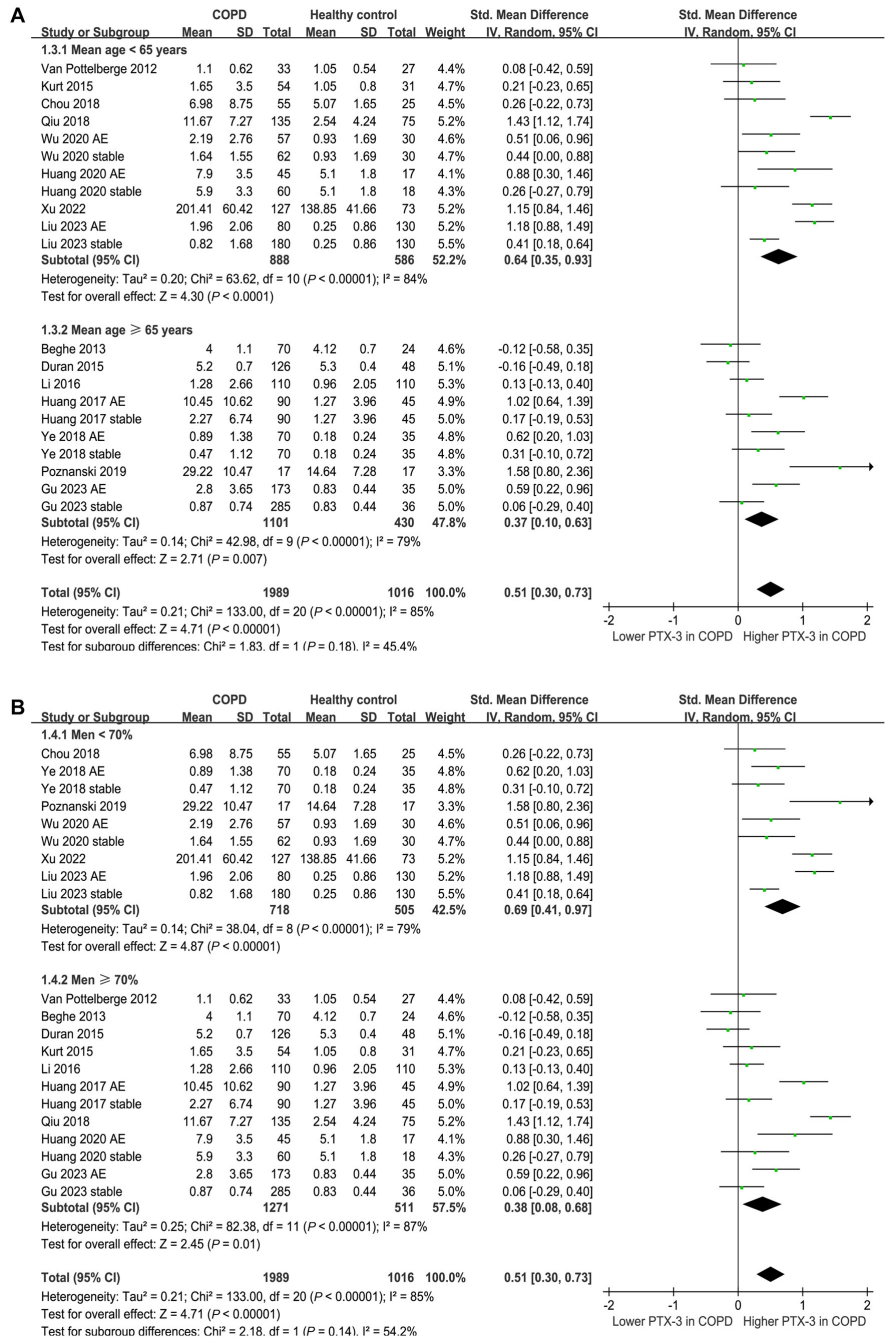
**Forest plots for the subgroup analyses comparing serum PTX-3 between patients with COPD and healthy controls; (A) subgroup analysis according to the mean age of the subjects; (B) subgroup analysis according to the proportion of men.** PTX-3: Pentraxin-3; COPD: Chronic obstructive pulmonary disease.

**Figure 4.**
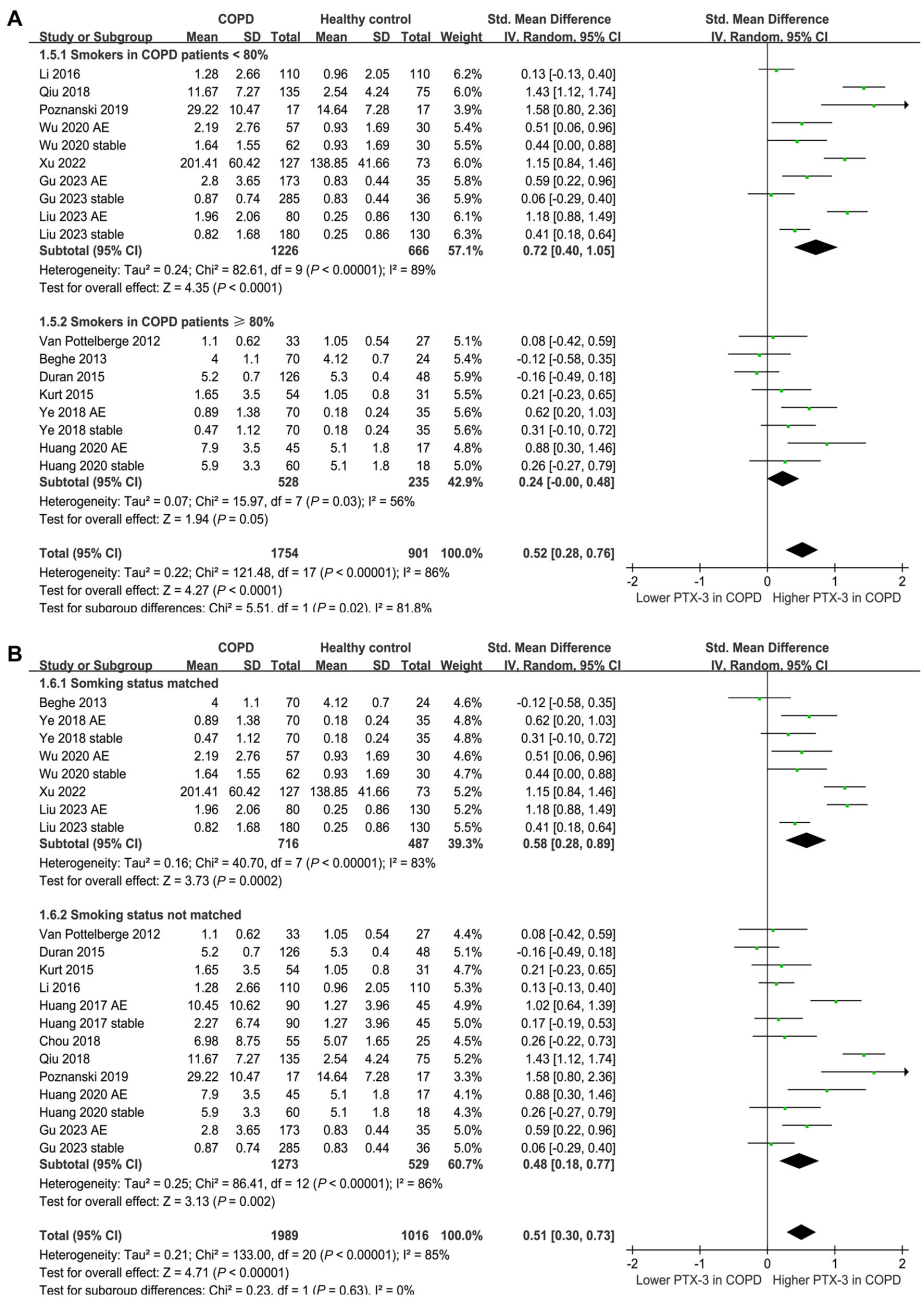
**Forest plots for the subgroup analyses comparing serum PTX-3 between patients with COPD and healthy controls; (A) subgroup analysis according to the proportion of smokers in COPD patients; (B) subgroup analysis according to whether the smoking status was matched between cases and controls.** PTX-3: Pentraxin-3; COPD: Chronic obstructive pulmonary disease.

Fifteen of the included studies [17–31] compared the serum levels of PTX-3 between patients with COPD and healthy controls. Because six of them reported the data of PTX-3 levels in patients with AECOPD and stable COPD separately [17, 19, 21, 22, 24, 25], these comparisons were included in the meta-analysis independently. The pooled results demonstrated a higher serum level of PTX-3 in patients with COPD compared to that in healthy controls (SMD: 0.51, 95% CI: 0.30–0.73, *P* < 0.001; *I*^2^ ═ 85%; Figure 2A). Subsequent subgroup analyses revealed consistent results across different patient groups. For patients with AECOPD vs stable COPD, the SMD were 0.71 and 0.36, respectively (*P* for subgroup difference ═ 0.11; Figure 2B). For studies with mean patient ages below 65 vs 65 years or older, the SMDs were 0.64 and 0.37, respectively (*P* for subgroup difference ═ 0.18; Figure 3A). For studies with proportions of men below 70% vs 70% or higher, the SMDs were 0.69 and 0.38, respectively (*P* for subgroup difference ═ 0.14; Figure 3B). Additionally, subgroup analyses showed similar results for studies of COPD patients with less than 80% smokers vs 80% or more smokers, with SMDs of 0.72 and 0.24, respectively (*P* for subgroup difference ═ 0.07; Figure 4A). Results were also similar in studies that matched smoking status between COPD patients and controls vs those that did not match, with SMDs of 0.58 and 0.48, respectively (*P* for subgroup difference ═ 0.63; Figure 4B). Furthermore, univariate meta-regression analyses indicated that the difference in serum PTX-3 levels between cases and controls was not significantly influenced by study characteristics, such as mean age, proportion of men, proportion of smokers in COPD patients, or study quality scores (all *P* values > 0.05; Table 3).

**Table 3:** Results of meta-regression analysis for the difference of serum PTX-3 between patients with COPD and healthy controls

**Variables**	**SMD for the difference of serum PTX-3 levels**		
	**Coefficient**	**95% CI**	***P* values**
Mean age (years)	−0.037	−0.094–0.021	0.20
Men (%)	−0.017	−0.037–0.004	0.11
Smokers in COPD group (%)	−0.0064	−0.0183–0.0055	0.27
NOS	−0.024	−0.393–0.344	0.89

COPD: Chronic obstructive pulmonary disease; PTX-3: Pentraxin-3; NOS: Newcastle–Ottawa Scale; SMD: Standardized mean difference; CI: Confidence interval.

### Difference in serum PTX-3 levels between patients with AECOPD and stable COPD

**Figure 5.**
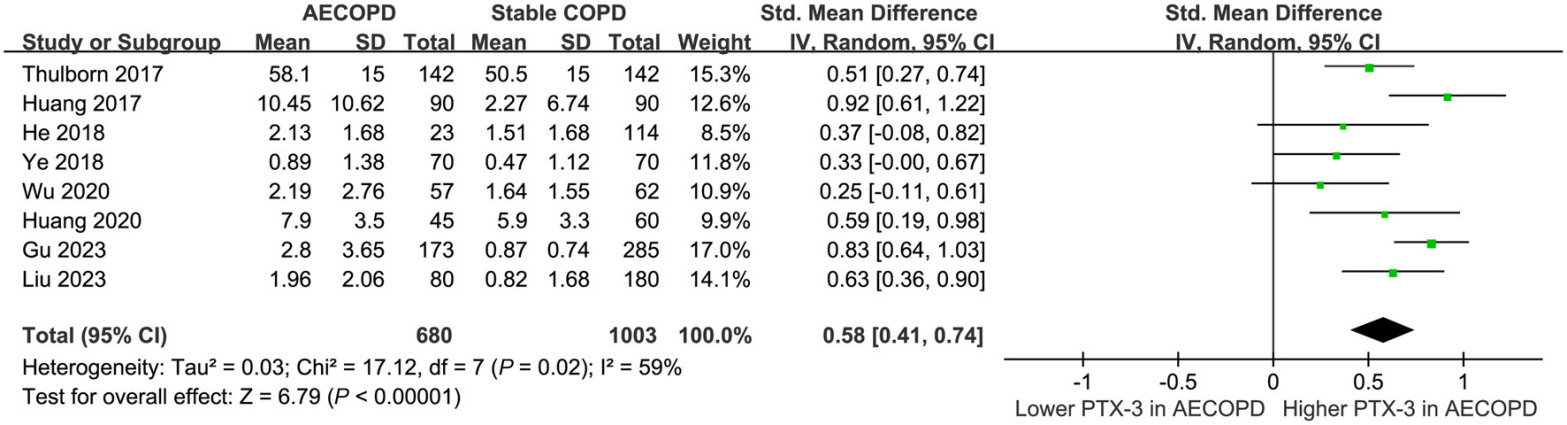
**Forest plots for the meta-analysis comparing serum PTX-3 between patients with AECOPD and stable COPD.** PTX-3: Pentraxin-3; COPD: Chronic obstructive pulmonary disease; AECOPD: Acute exacerbations of COPD.

Eight studies [17, 19, 21, 22, 24, 25, 37, 38] directly compared the serum levels of PTX-3 between patients with AECOPD and stable COPD. The pooled results showed a higher serum level of PTX-3 in patients with AECOPD compared to those with stable COPD (SMD: 0.58, 95% CI: 0.41–0.74, *P* < 0.001; *I*^2^ ═ 59%; Figure 5).

### Publication bias evaluation

**Figure 6.**
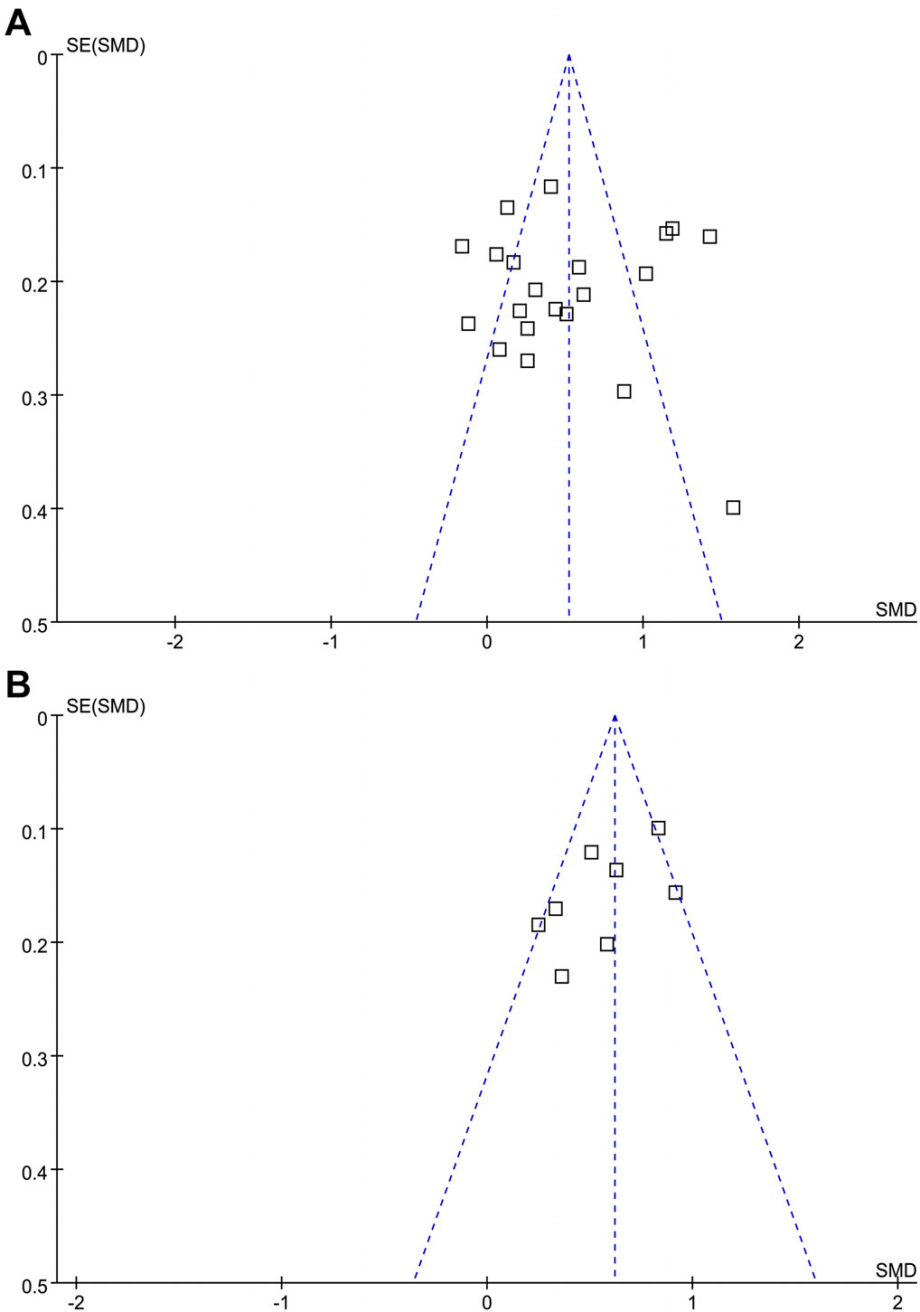
**Funnel plots for the meta-analyses; A, funnel plots for the meta-analysis comparing serum PTX-3 between patients with COPD and healthy controls; and B, funnel plots for the meta-analysis comparing serum PTX-3 between patients with AECOPD and stable COPD.** PTX-3: Pentraxin-3; COPD: Chronic obstructive pulmonary disease; AECOPD: Acute exacerbations of COPD.

Funnel plots for both primary and secondary outcomes appeared symmetrical, indicating a low risk of publication bias (Figure 6A and 6B). This was confirmed by Egger’s test, which showed nonsignificant results (*P* ═ 0.92 for the difference in PTX-3 levels between patients with COPD and controls; *P* ═ 0.41 for the difference in PTX-3 levels between patients with AECOPD and stable COPD).

## Discussion

This meta-analysis synthesizes data from 17 studies, demonstrating that serum PTX-3 levels are significantly higher in COPD patients compared to healthy controls, with even greater elevations observed in patients experiencing AECOPD. The pooled results show an SMD of 0.51 for PTX-3 levels between COPD patients and healthy controls and an SMD of 0.58 between AECOPD and stable COPD patients. These findings indicate that PTX-3 could be a reliable biomarker for both COPD presence and exacerbation severity.

To the best of our knowledge, this study is the first meta-analysis that evaluated the different serum levels of PTX-3 between patients with COPD and healthy controls. The increased serum PTX-3 levels in COPD and AECOPD patients are linked to the protein’s role in the innate immune response and inflammation [39]. PTX-3 is produced in response to pro-inflammatory cytokines, microbial components, and tissue damage. In COPD, chronic exposure to irritants like cigarette smoke triggers a persistent inflammatory state, leading to elevated PTX-3 production [13]. PTX-3 regulates inflammation by recognizing pathogens, activating the complement system, and modulating leukocyte recruitment. It binds to microbial components and apoptotic cells, facilitating their recognition by phagocytes and enhancing pathogen clearance [40, 41]. PTX-3 also interacts with complement pathway components, such as C1q, enhancing opsonization and phagocytosis [40–42]. This multifaceted role in modulating immune responses and inflammation highlights PTX-3’s involvement in COPD pathogenesis, where continuous tissue damage and repair are critical. During acute COPD exacerbations, often triggered by infections or environmental pollutants, there is a further surge in inflammatory mediators, resulting in even higher PTX-3 levels. Studies have linked elevated serum PTX-3 or certain PTX-3 polymorphisms with invasive pulmonary aspergillosis [43, 44] or pulmonary fungal disease [45]. These exacerbations are characterized by increased airway inflammation, heightened systemic inflammation, and rapid lung function decline. The acute phase response, marked by upregulated PTX-3, plays a crucial role in amplifying the inflammatory cascade during these episodes [46]. This elevation in PTX-3 during AECOPD underscores its potential use in distinguishing between stable and exacerbated disease states.

Subgroup and meta-regression analyses within this meta-analysis demonstrated that the differences in PTX-3 levels between COPD patients and healthy controls, as well as between AECOPD and stable COPD patients, are consistent across various demographic and clinical characteristics. These analyses revealed no significant impact of age, sex, or smoking status on PTX-3 levels, underscoring the robustness of PTX-3 as a biomarker across diverse patient populations.

This meta-analysis has several strengths, including a comprehensive search strategy, stringent inclusion criteria, and the utilization of standardized tools for quality assessment and data extraction. The large sample size and inclusion of studies from various geographic locations enhance the generalizability of the findings. However, some limitations should be noted. Significant heterogeneity was observed among the included studies, possibly due to differences in study design, population characteristics, and measurement techniques. Although multiple subgroup and meta-regression analyses were conducted, the source of heterogeneity remains unclear. Despite employing a random-effects model to account for this heterogeneity, it remains a potential source of bias. Additionally, all included studies were of a case-control design, precluding the determination of a prospective association between elevated serum PTX-3 and the development and exacerbation of COPD. Moreover, although age, sex, BMI, and smoking were matched to varying extents between COPD patients and controls, other confounding factors may still exist. High-quality prospective studies with adequately adjusted confounding factors are required to validate the results of this meta-analysis.

The findings of the meta-analysis suggest that serum PTX-3 could serve as a valuable biomarker for COPD diagnosis and monitoring, as well as for assessing the severity of acute exacerbations. Elevated PTX-3 levels in COPD patients, particularly during exacerbations, could aid clinicians in identifying high-risk patients and tailoring treatment strategies accordingly. Integrating PTX-3 measurements into routine clinical practice could improve COPD management by enabling more precise monitoring of disease progression and response to therapy. Future research should focus on large-scale, longitudinal studies to validate the role of PTX-3 as a biomarker for COPD and its exacerbations. Investigating the mechanisms underlying PTX-3 production and its interactions with other inflammatory mediators in COPD could provide deeper insights into the disease’s pathophysiology. Additionally, exploring the effects of interventions, such as anti-inflammatory treatments, on PTX-3 levels could inform therapeutic strategies aimed at modulating the inflammatory response in COPD.

## Conclusion

In conclusion, this meta-analysis reveals that serum PTX-3 levels are significantly higher in COPD patients compared to healthy controls, with even greater elevations during acute exacerbations. These findings highlight the potential of PTX-3 as a biomarker for COPD diagnosis and for evaluating exacerbation severity. Although some limitations exist, the consistent results across different subgroups underscore the clinical significance of PTX-3. Further research is needed to validate these findings and explore the therapeutic potential of targeting PTX-3 in the management of patients with COPD.

## Data Availability

All the data generated during the study was within the manuscript.
